# Ultrastrong Exciton–Photon Coupling in Broadband Solar Absorbers

**DOI:** 10.1021/acs.jpclett.1c02898

**Published:** 2021-10-28

**Authors:** Clara Bujalance, Victoria Esteso, Laura Caliò, Giulia Lavarda, Tomás Torres, Johannes Feist, Francisco José García-Vidal, Giovanni Bottari, Hernán Míguez

**Affiliations:** † Multifunctional Optical Materials Group, Institute of Materials Science of Sevilla, Consejo Superior de Investigaciones Científicas−Universidad de Sevilla (CSIC-US), Américo Vespucio 49, 41092 Sevilla, Spain; ‡ Departamento de Química Orgánica, Universidad Autónoma de Madrid, 28049 Madrid, Spain; § IMDEA Nanociencia, Campus de Cantoblanco, 28049 Madrid, Spain; ∥ Institute for Advanced Research in Chemical Sciences (IAdChem), Universidad Autónoma de Madrid, 28049 Madrid, Spain; ⊥ Departamento de Física Teórica de la Materia Condensada and Condensed Matter Physics Center (IFIMAC), Universidad Autónoma de Madrid, 28049 Madrid, Spain

## Abstract

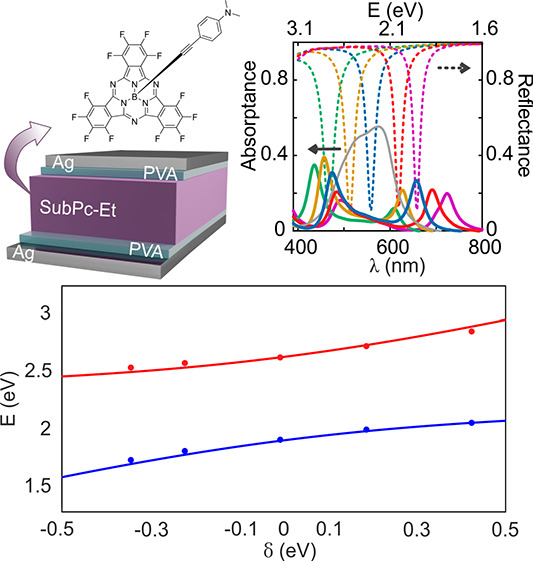

The recent development of organic polaritonic solar cells, in which sunlight absorbers and photon modes of a resonator are hybridized as a result of their strong coupling, has revealed the potential this interaction offers to control and enhance the performance of these devices. In this approach, the photovoltaic cell is built in such a way that it also behaves as an optical cavity supporting spectrally well-defined resonances, which match the broad absorption bands of the dyes employed. Herein we focus on the experimental and theoretical analysis of the specific spectral and angular optical absorption characteristics of a broadband light harvester, namely a subphthalocyanine, when operating in the ultrastrong coupling regime. We discuss the implications of having a broad distribution of oscillator strengths and demonstrate that rational design of the layered structure is needed to optimize both the spectral and the angular response of the sunlight harvester dye.

An organic compound strongly coupled to an optical cavity^1^ can intensely absorb light at frequencies for which its intrinsic absorption is practically none^2^ or emit light at spectral ranges at which the uncoupled system barely shows optical activity.^3^ These effects arise as a result of the reconfiguration of the electronic structure of the molecules and the optical modes of the cavity caused by their strong coupling.^4−6^ In this regime, the eigenmodes of the ensemble must be described as hybrid light–matter states, also known as polaritons. Following an intense activity focused on the description of its fundamental aspects, this phenomenon has been put into practice aiming at developing a polariton-based technology^7^ that takes advantage of the possibilities that the new electronic and optical properties of hybrid light–matter states open. Hence, its potential has been proven in fields like lasing,^8−10^ photodetection,^11^ catalysis,^12,13^ photochemistry,^14−16^ and, from an even broader perspective, in synthetic chemistry, where novel reaction pathways are being explored.^16−19^

Recently, the spectral control over the polaritonic absorption of strongly coupled organic compounds has been used to reduce photon energy losses in organic solar cells by effectively diminishing the bandgap of the absorber while, at the same time, decreasing the electron driving force and hence charge transfer losses.^20^ For this purpose, a layered structure made of subphthalocyanine (SubPc)-based thin films, each one performing a different function (electron donor or acceptor materials), was sandwiched between two metal contacts, which played the role of both electrical contacts and mirrors. One specific characteristic of the strong-coupling configuration employed in solar cells is the inhomogeneous character of the electronic transitions involved in the coupling: the absorption bands involved are either excitonic Q-bands, characteristic of porphyrinoids and described by Gouterman’s model,^21,22^ which results from the convolution of several HOMO–LUMO transitions, or charge transfer (CT) bands, which are usually even broader. Recently, we showed that the specific characteristics of CT bands of a SubPc made them more prone to yield *weak coupling* when interacting with optical cavity modes, while the more intense and narrow SubPc Q-bands tend to favor the formation of polaritons, characteristic of *strong coupling*.^23^ However, how to deal with the design of a polaritonic light harvesting system based on a broadband absorber operating under the *ultrastrong coupling* regime is still an open question, a few relevant examples being found in the literature.^2,11,24−27^ The equations commonly employed to predict the response of a polaritonic system assume narrow electronic transitions with a well-defined spectral position and oscillator strength, while solar dyes are inherently inhomogeneous broadband absorbers. Another relevant aspect for energy conversion applications is how the unwanted parasitic absorption that occurs in the metallic mirror/contacts is affected by the polaritonic interaction.

In this work, we analyze the absorption properties of a broadband solar molecular absorber, a perfluorinated SubPc substituted with an ethynylaniline moiety in its axial position (thereafter referred to as SubPc-Et, Figure 1a),^28^ embedded in an optical cavity and undergoing ultrastrong exciton–photon coupling. To do so, we have built resonators that resemble the characteristics of recently reported photovoltaic devices^20^ in which strong light–matter interactions have opened new routes to control their performance. We address the question of dealing with an inhomogeneous distribution of oscillators instead of a well-defined single electronic transition and identify the best way to define reference values for the optical and excitonic transitions that partake in the hybrid light–matter polaritonic modes. Moreover, we find that unlike most other absorption control or enhancement approaches based on photonic resonances, ultrastrong exciton–photon coupling may be designed to yield an almost flat angular response, which, as we prove, results from the balance of the opposite trends shown by the S and P polarization modes. Furthermore, modeling allows us to quantify the parasitic absorption occurring at the metallic contacts and confirm that their angular and spectral dispersion resembles that of polaritonic modes.

**Figure 1:**
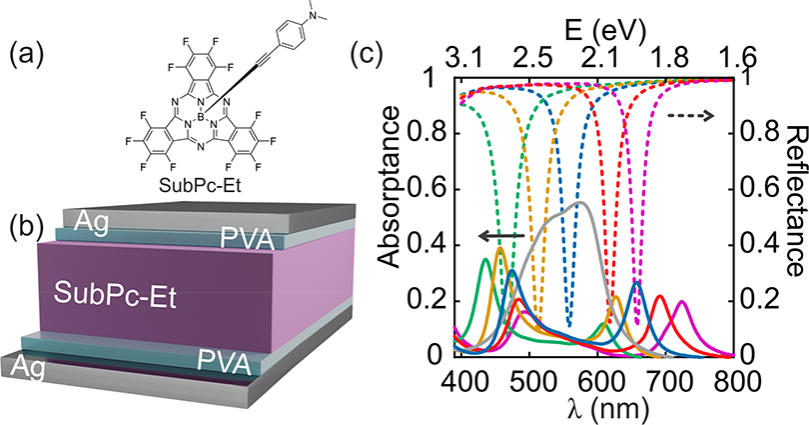
(a) Chemical structure of the SubPc derivative used in this work, attained by anchoring an ethynylaniline ligand to the boron atom of a perfluorinated SubPc. Synthetic details are available in ref (28). (b) Schematic representation of the structure of the Fabry–Pérot resonators prepared for this work. (c) Absorptance spectra (colored solid lines) of resonators built varying the cavity thickness, namely, 100 nm (green), 115 nm (yellow), 130 nm (blue), 150 nm (red), and 163 nm (purple). The calculated normal incidence reflectance spectra of the corresponding underlying optical cavities are plotted following the same color code (dashed lines). For the sake of comparison, the absorptance of a bare SubPc-Et film is also shown (solid light gray line).

A set of metallic optical resonators embedding films of different thickness made of SubPc-Et molecules were prepared following a previously reported procedure.^23^ In brief, the cavities consist in a layer of SubPc-Et sandwiched between two poly(vinyl alcohol) (PVA) thin films (18–25 nm), which are in turn coated by two silver mirrors (35 nm). The architecture of the resonators built for this work is drawn in Figures 1b. Bare SubPc-Et films deposited on glass display an intense Q-band absorption in the visible region. In Figure 1c, we show a representative normal incidence absorptance of a 94 nm thick SubPc-Et film (light gray line). Once integrated in a metallic optical cavity, the absorptance of the ensemble presents characteristic polaritonic traits, with two well-defined absorption maxima separated by an energy gap. Colored solid lines in Figure 1c correspond to the spectra of optical cavities of different thickness (determined by the widths of the SubPc-Et and the two surrounding PVA films), namely, 100 nm (green), 115 nm (yellow), 130 nm (blue), 150 nm (red), and 163 nm (purple). The corresponding SubPc-Et thickness in each case is 50, 71, 94, 106, 113, and 146 nm. The calculated normal incidence reflectance spectra of the corresponding underlying (not absorbing and with effective refractive index *n* = 1.56, a choice that will be justified below) optical cavities are plotted following the same color code (dashed lines in Figure 1c), the first-order resonant photon modes being readily identified as well-defined minima.

The splitting of the absorption spectrum reflects the opening of an energy gap as a result of the anticrossing of the upper and lower polaritonic branches. This can be seen in the energy dispersion relations plotted in Figures 2a,b, which corresponds to a resonator with thickness of 130 nm (similar results are attained for all remaining optical cavities and are included in the Supporting Information; see Figures S1–S4). These maps are obtained from angle and polarization dependence measurements of both reflectance (*R*, Figures 2c,d) and transmittance (*T*, Figures 2e,f), from which absorptance (*A* = 1 – *R* – *T*) is attained. A double-goniometer configuration (Universal Measurement Accessory attached to a Cary5000 spectrophotometer) was used for this characterization, in which sample and detector can be independently rotated, and hence arbitrary incident and collection angles may be selected. *A*, *R*, and *T* intensity maps are plotted versus both the photon energy (*y*-axis) and the parallel component of the wavevector  (*x*-axis, ) following a standard representation. The absorption gap opening arises from the anticrossing of the upper and lower polaritonic energy branches, and its width is given by ℏΩ_R_, where ℏ is the reduced Planck constant and Ω_R_ is the Rabi frequency, which stands for the rate at which energy is exchanged between the photonic mode and the electronic transition. The exciton–photon coupling strength may be estimated through the ratio Ω_R_/ω_0_,^29^ which compares the polariton energy gap with the intrinsic material transition energy (ω_0_). Polaritonic gaps for the set of resonators are estimated by measuring the energy jump at the  point where the cavity mode, ω_cav_, matches the frequency of the electronic transition, ω_0_. In the case of the cavity displayed in Figure 2, this occurs for  ≈ 0, where the cavity resonance dispersion curve (white dotted line) intersects the horizontal line, indicating the spectral position of the electronic transition (red dotted line). This yields ℏΩ̵_R_ ≈ 750 meV, corresponding to Ω_R_/ω_0_ ≈ 0.38, well above the threshold usually set to consider an exciton–photon system to be in the ultrastrong coupling regime (Ω_R_/ω_0_ > 0.2).^29^ Note that films of SubPc-Et present a stronger Q-band absorption (i.e., the electronic transitions involved have larger oscillator strengths) and are of higher optical quality (i.e., they show more uniformity and less scattering) than those made of the SubPc molecules we employed to study the different coupling between CT and Q-bands in a previous work.^23^ In that case, the analysis of the coupling between the Q-band and the cavity mode yielded Ω_R_/ω_0_ ≈ 0.036, an order of magnitude smaller than in the present case.

**Figure 2:**
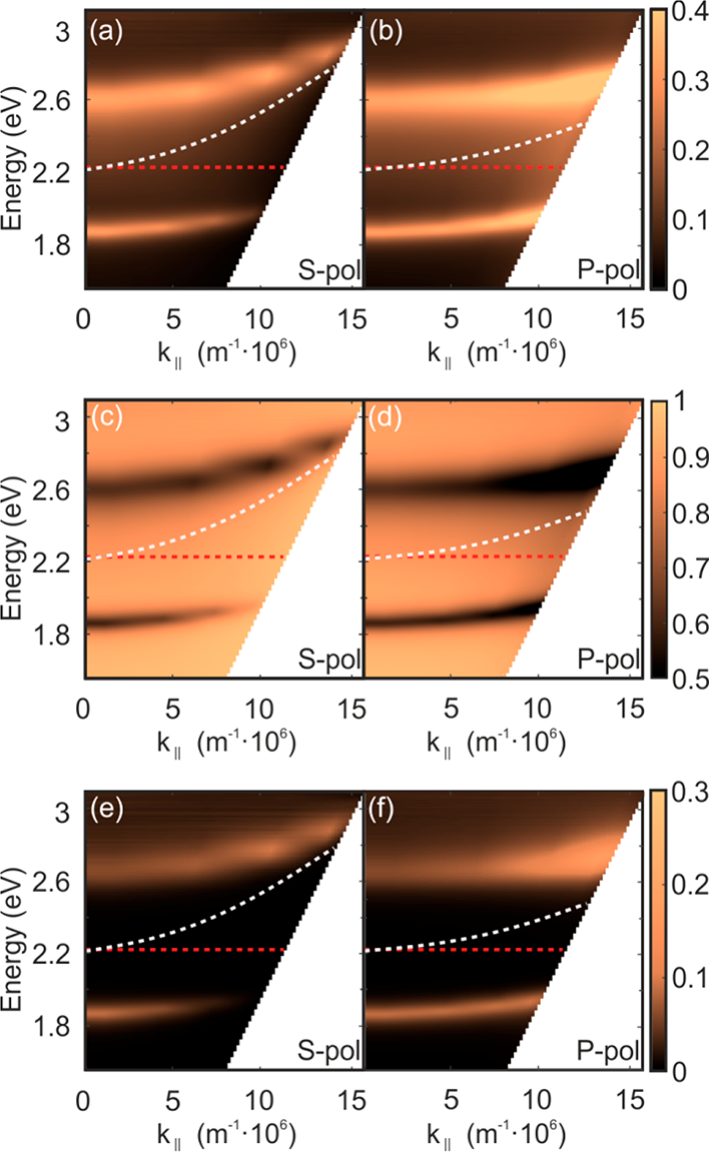
Dispersion curves attained from the angular dependence measurements of the absorptance (a, b), reflectance (c, d), and transmittance (e, f) of an optical resonator made of a 94 nm thick SubPc-Et film (total cavity thickness 130 nm, after adding the thickness of the PVA leveling layers). Left and right panels correspond respectively to S-polarized (or *transversal electric*, TE, i.e., electric field *E* perpendicular to the plane of incidence) and P-polarized incident light (or *transversal magnetic*, TM, with *E* contained in the plane of incidence). The white dashed line corresponds to the underlying cavity first-order mode dispersion, while the red dashed horizontal line indicates the spectral position of the SubPc-Et absorption outside the cavity taken as reference.

It is worth comparing our results with previous ones for other organic metallic cavities. Kéna-Cohen and co-workers recently demonstrated a coupling ratio of Ω_R_/ω_0_ ≈ 0.48^11^ for a ultrastrongly coupled mixed phthalocyanine/C_60_ film as well as a remarkable Ω_R_/ω_0_ ≈ 0.62 for a polymethine dye.^26^ Also, Sanvitto, Gigli, and co-workers achieved Ω_R_/ω_0_ ≈ 0.60 for a squaraine-filled microcavity.^2^ These values are among the highest reported for organic materials, in both cases deposited by evaporation techniques, embedded in Fabry–Pérot cavities. The strongly coupled SubPc film herein presented holds the highest Ω_R_/ω_0_ for a solution processed organic material.

To rationalize the main spectral features observed for the different cavities herein analyzed, a model is required. The spectral positions at which the upper (ω_+_) and lower (ω_–_) polaritonic absorption peaks occur can be approximated by the following expression, which results from the diagonalization of the Jaynes–Cummings Hamiltonian:^6^
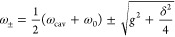
1where *g* is the coupling constant and δ is the detuning between ω_cav_ and ω_0_, δ = ω_cav_ – ω_0_. Although eq 1 describes a strongly coupled system of two modes with well-defined frequencies, it has been theoretically proven^30^ and experimentally confirmed^2,25^ that a similar description holds for broadband absorbers. However, using this model for an inhomogeneously broadened absorption peak requires an adequate approximation of both ω_cav_ and ω_0_. To do so, we have determined the optical constants of the bare SubPc-Et film deposited on a quartz substrate. We have employed the method developed by Forouhi and Bloomer,^31^ based on the fitting of the experimental reflectance and transmittance measured at different incidence angles and polarizations. This method considers a series of Lorentz oscillators, each one of them characterized by an oscillator strength and width, which account for the different electronic transitions contributing to the absorption band under analysis. In Figure 3a, we plot the experimental absorptance of the SubPc-Et film (blue line) and the best fitting (red dashed line) as well as the corresponding oscillator curves employed for the fitting (light gray lines). This deconvolution is arbitrary insofar as the theoretical study of the electronic structure and optical properties of this specific SubPc has not been reported, and the number of electronic transitions involved in the formation of the Q-band varies as a function of the axial and peripheral ligands present in the molecule. In any case, this empirical approach allows us to establish a criterion to define a reference excitonic transition frequency (i.e., ω_0_), which we set as the average value of the spectral position of the oscillator maxima, weighted by the area of each peak. This value (λ_0_ = 557 nm, ω_0_ = 2.23 eV) is indicated with a vertical dark gray dash-dotted line in Figure 3a. Also, both real and imaginary components, *n*(λ) and κ(λ), of the refractive index of the SubPc-Et film attained by using the Forouhi–Bloomer model are displayed in Figure 3b. From these curves, the representative effective (constant) refractive index of the underlying, nonabsorbing, optical cavity is attained by averaging the real part of the refractive index of the SubPc-Et film in the region of interest (400 nm < λ < 800 nm). This value, *n* = 1.56, is highlighted with a horizontal dark gray dash-dotted line in Figure 3b, and it is the one we use to calculate ω_cav_. Once we have rationally estimated ω_0_ and ω_cav_, we are in a position to compare our data with the expected theoretical dispersion given by eq 1. Figure 3c shows both the experimental (red and blue circles) and theoretical (solid lines) dependence of the spectral position of the upper (ω_+_, red colored) and lower (ω_–_, blue colored) polaritonic branches versus the degree of detuning δ, estimated from the above-mentioned values of ω_0_ and ω_cav_. It should be noticed that this choice of ω_0_ and ω_cav_ allows for the best correspondence between theory and experiment. Other options were tested and showed a larger discrepancy between both data sets. Each pair of scatters corresponds to the spectral position of the upper and lower polaritonic branches extracted from the absorptance spectra of each cavity measured at normal incidence.

**Figure 3:**
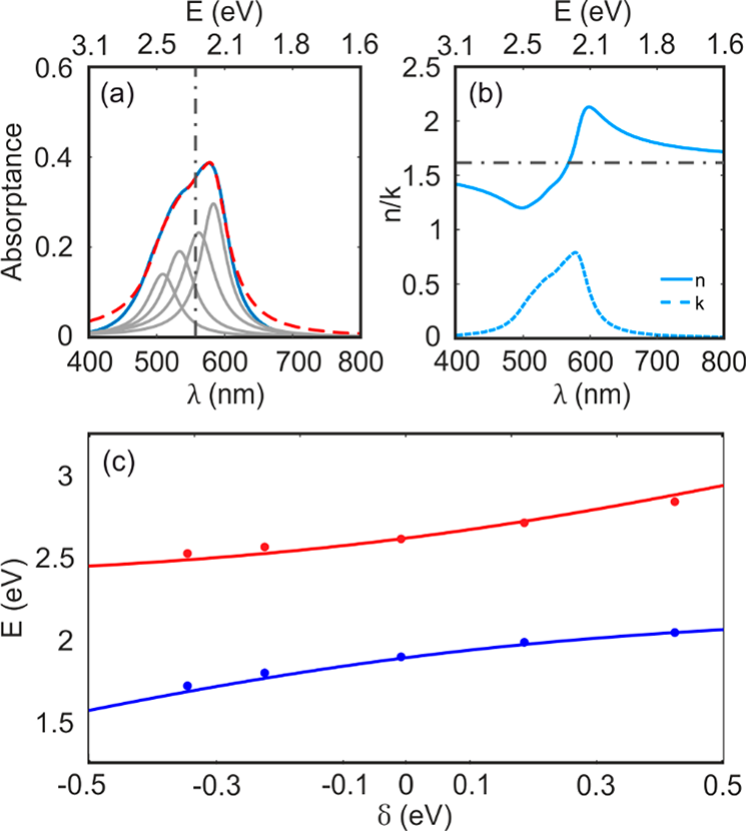
(a) Experimental (blue line) and fitted (red dashed line) absorptance of a SubPc-Et film. The contributions from the different oscillators considered for the fitting are plotted as light gray lines. The vertical dark gray dash-dotted line corresponds to the average value of the spectral position of the oscillator maxima, weighted by the area of each peak, which we take as ω_0_. (b) The real (solid line) and imaginary (dashed line) parts of the refractive index, *n* and *k*, estimated from the fitting of the optical properties of the SubPc-Et film. The horizontal dark gray dash-dotted line indicates the average value of *n* in the region of interest (400 nm < λ < 800 nm), which we take as the refractive index of the underlying, nonabsorbing, optical medium. (c) Experimental (scatter graphs) and theoretical (solid lines) dependence of the spectral position of the upper (ω_+_, red colored) and lower (ω_–_, blue colored) polaritonic branches versus the degree of detuning δ, estimated from the above-mentioned values of ω_0_ and ω_cav_.

The fact that metallic mirrors are used to make the optical cavity implies that parasitic absorption (i.e., nonproductive from the point of view of light harvesting) may be present. To analyze how significant this effect is in an ultrastrongly coupled light harvester, we follow a classical electrodynamics approach, described in detail in ref (3) and based on the transfer matrix method. This allows us to discriminate the absorption that occurs at each layer in the assembly. In Figures 4a and 4b, we show the calculated spectral and spatial profiles of the light electric field intensity, |**E**(**r**)|^2^, and the normalized absorptance per unit length, δ*A*, respectively, for the same cavity chosen as illustrative example in Figure 2. These intensity maps have been attained considering a plane wave impinging normally on the resonators (= 0). They permit not only visualizing the first-order resonant mode splitting, which is at the origin of the polariton absorption energy gap, but also estimating the absorptance spectra of each layer in the assembly. This is represented in Figure 4c. Note that the actual absorption taking place in the SubPc-Et film (purple solid line) is slightly smaller than the total one (red solid line) due to the presence of optical losses in the top (dark gray line) and bottom (light gray line) silver mirrors. Total parasitic absorptance is also plotted (black solid line). A comparison between the areas below these curves indicates that the losses in the metallic mirrors amount for 20.1% of the total absorption of the device. A similar analysis performed at different incidence angles allows us to obtain the energy dispersion of both the SubPc-Et (productive) and the metals (parasitic) absorptance, which are plotted as intensity maps in Figures 4d and 4e (results for nonpolarized light, estimated by averaging the experimental results attained for the S and P polarizations at each incidence angle). As expected, the absorption occurring at the metal layers follows the same dispersion than the polaritonic branches, since incident light can only couple to the cavity via the polaritonic modes. A rigorous analysis of the effect of the coupling (be it weak, strong, or ultrastrong) of a light harvesting material to metallic cavities should always include an estimation of contributions of the mirrors to the absorption. In this case, as it can be concluded from the comparison of Figures 2a and 2b and Figure 4e, optical losses in the mirrors only modify quantitatively, and by a small amount, the experimental polaritonic energy dispersion attained.

**Figure 4:**
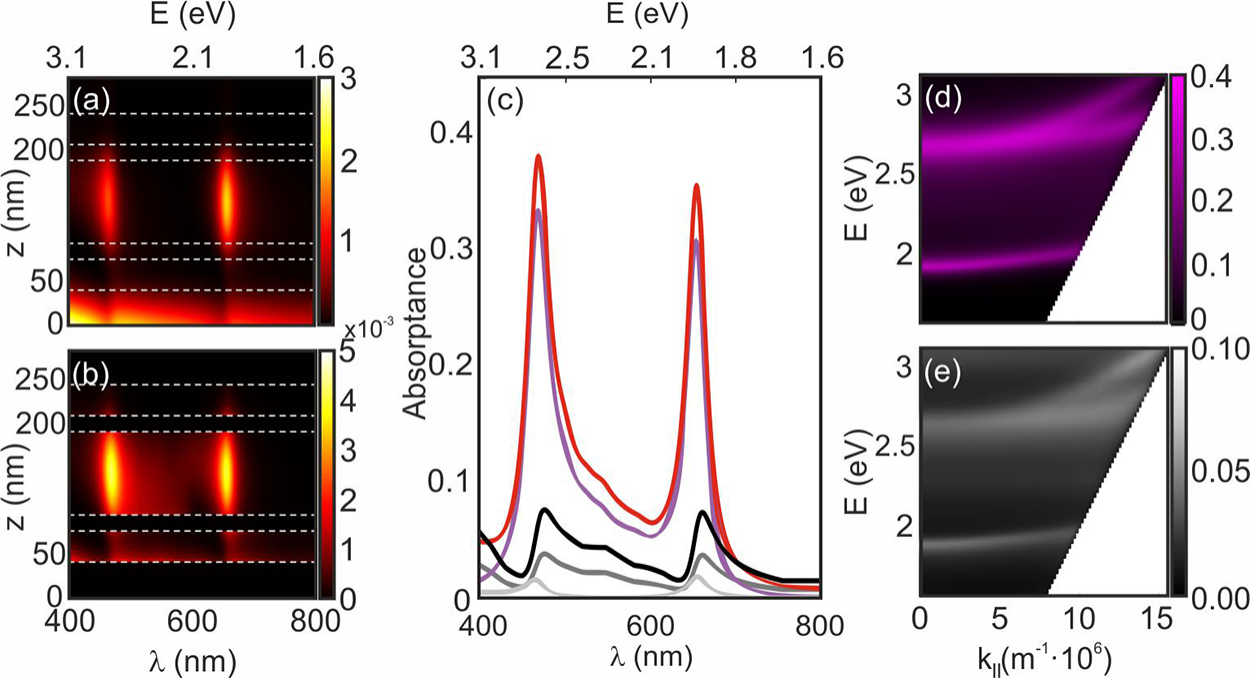
Calculated spatial and spectral profiles of (a) the electric field intensity |**E**(**r**)|^2^ and (b) the absorbed luminous power *P*_*A*_ for a SubPc-Et cavity of thickness 130 nm, at normal incidence. (c) Discriminated absorptance at normal incidence of each layer in the cavity, namely, the SubPc-Et film (purple line) and top (dark gray) and bottom (light gray) silver mirrors. Total optical loss spectrum in the metals (black) and the full absorptance of the cavity (red) are also plotted. Intensity maps in (d) and (e) show the energy dispersion of both the SubPc-Et (productive) and the metals (parasitic) absorptance of nonpolarized light.

Next, we evaluate the specific features of solar absorption in an ultrastrongly coupled polaritonic light harvester. To do so, we calculated the solar spectrum weighted absorptance (SSWA) that results from multiplying our experimental data by the (normalized) AM1.5 solar irradiance spectrum standard, which accounts for sunlight rays impinging on the earth’s surface and traveling through an air mass equivalent to that of 1.5 atm. Also, we calculate the normalized wavelength integrated SSWA at each angle of incidence using the expression
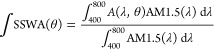
2The results are displayed in Figure 5, where we plot the SSWA attained at different angles (Figures 5a–c) and the ∫SSWA(θ) (Figures 5d–f) for S-polarized (a, d), P-polarized (b, e), and nonpolarized (c, f) light. Note that even when the integrated absorption estimated for S- and P-polarized incident light shows a significant angular dependence, the results for nonpolarized light present an almost flat response for both productive and parasitic absorptions, as shown in Figure 5f. This low dispersion is the result of the balance between the opposite trends observed for the sunlight absorption intensities displayed by S and P polarizations. The same analysis of ∫SSWA(θ) is performed in the remaining optical cavities (Figures S5–S8), finding that the larger the absolute value of δ, the more significant the angular dependence for nonpolarized light (see Figure S9, in which this effect can be readily seen). The flat response of ultrastrongly coupled configurations has interesting consequences from the point of view of the application of these structures in light-to-energy conversion devices, as one of the main drawbacks when photonic designs are implemented in solar cells, photodetectors, or light-emitting devices is the introduction of unwanted strong angular dependences that may limit their utility.

**Figure 5:**
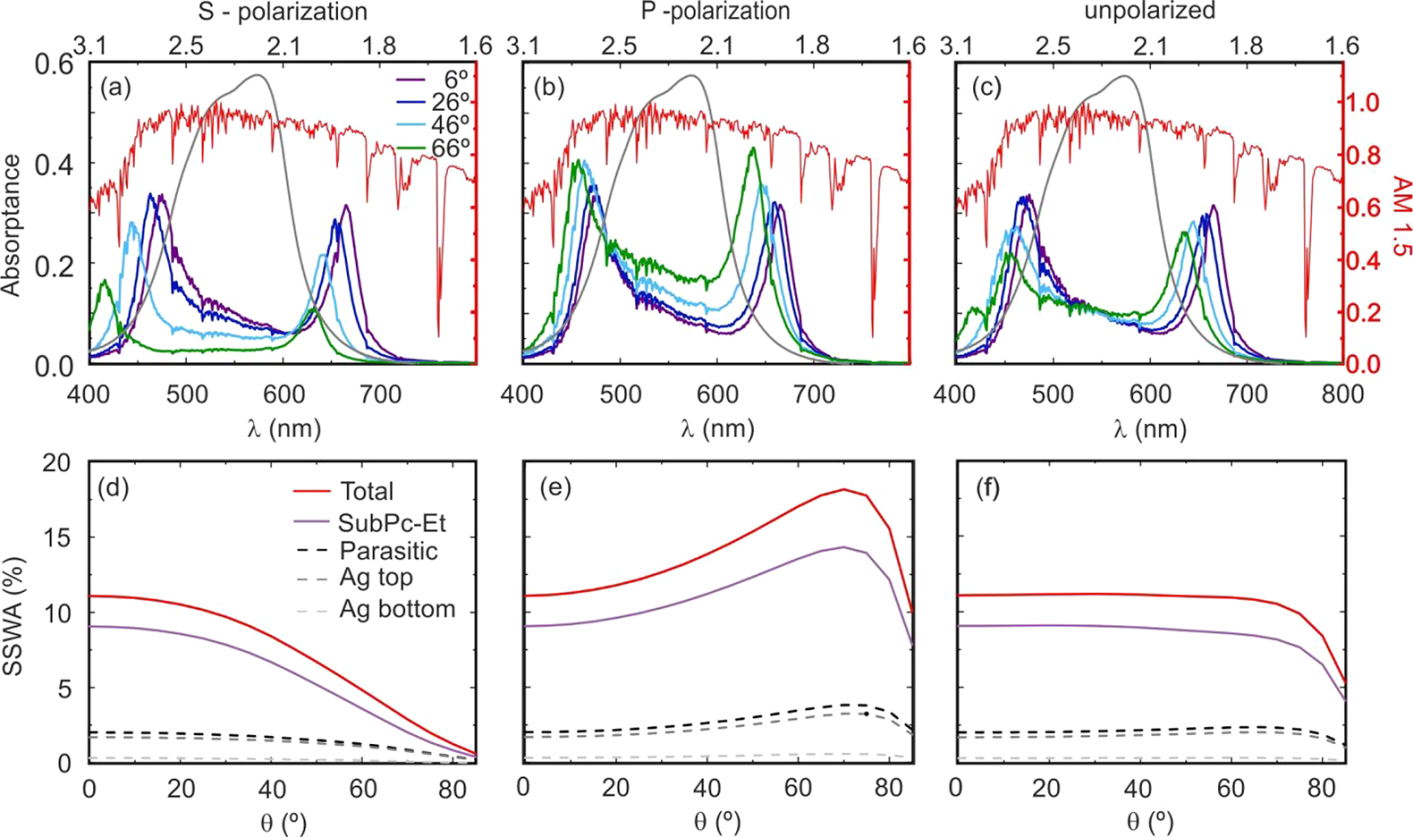
(a–c) Solar spectrum (AM1.5) weighted absorptance, SSWA, of a metallic optical cavity embedding a 94 nm thick SubPc-Et film sandwiched between two PVA thin films. Results are shown for (a) S-polarized, (b) P-polarized, and (c) nonpolarized light incident at angles of 6° (purple line), 26° (blue), 46° (light blue), and 66° (green) with respect to the cavity surface normal. The normalized AM1.5 spectrum and the absorptance of a bare SubPc-Et film are also plotted (red and gray solid lines, respectively). (d–f) Wavelength-integrated SSWA, ∫SSWA, versus angle of incidence of sunlight for each one of the layers in the cavity, namely, the SubPc-Et film (purple line), top mirror (dark gray dashed line), and bottom mirror (light gray dashed line). Total integrated parasitic absorption (black dashed line) and full integrated absorption of the cavity (red solid line) are also plotted. Results are shown for (d) S-polarized, (e) P-polarized, and (f) nonpolarized light.

In summary, we have performed a systematic analysis of the light harvesting properties of a canonical broadband solar molecular absorber of interest in photovoltaic devices, namely a subphthalocyanine, when it is ultrastrongly coupled to an optical cavity. We have tackled the problem of dealing with a broadband absorber and proposed a way to attain the parameters that label the optical and excitonic transitions involved in the polaritonic interaction. We demonstrate that this approach allows employing standard models to describe and design the exciton–photon polariton modes of the resonator. Finally, we have studied in detail the spectral and angular response of the resonator acting as a sunlight harvester, showing that light harvesting metallic cavities can be designed in a such a way that both productive and parasitic absorption present an almost dispersionless response when irradiated with nonpolarized light, a result of the balance between the opposite behaviors displayed by S and P polarizations. We believe our results are of relevance for further development of polaritonic light harvesting devices (solar cells and photodetectors).

## Supporting Information Available

Dispersion curves attained from the angular dependence measurements of the absorptance, reflectance and transmittance, and wavelength integrated SSWA versus angle of incidence of sunlight for each one of the layers comprising the optical resonator for the cavity thicknesses considered in the text (PDF)
