# Supplementary material for: Ultrastrong Exciton–Photon Coupling in Broadband Solar Absorbers
Source: J Phys Chem Lett. 2021 Oct 28;12(43):10706–12. doi: 10.1021/acs.jpclett.1c02898 (PMC8573775; doi:10.1021/acs.jpclett.1c02898)
Supplement: Supplementary file 1 — jz1c02898_si_001.pdf [file jz1c02898_si_001.pdf]

Supporting Information

Ultra-strong Exciton-photon Coupling of Broad Band Solar Absorbers.

*Clara Bujalance,[1 §] Victoria Esteso,[1 §] Laura Caliò,[1 §] Giulia Lavarda,[2] Tomás Torres,[2,3,4] Johannes Feist,[5] Francisco José García-Vidal,[5] Giovanni Bottari,[2,3,4 *] Hernán Míguez[1 *]*

[1] Multifunctional Optical Materials Group, Institute of Materials Science of Sevilla, Consejo Superior de Investigaciones Científicas – Universidad de Sevilla (CSIC-US), Américo Vespucio 49, 41092, Sevilla, Spain.

[2] Departamento de Química Orgánica, Universidad Autónoma de Madrid, 28049 Madrid, Spain.

[3] IMDEA-Nanociencia, Campus de Cantoblanco, 28049 Madrid, Spain.

[4] Institute for Advanced Research in Chemical Sciences (IAdChem), Universidad Autónoma de Madrid, 28049 Madrid, Spain.

[5] Departamento de Física Teórica de la Materia Condensada and Condensed Matter Physics Center (IFIMAC), Universidad Autónoma de Madrid, 28049 Madrid, Spain

AUTHOR INFORMATION

Corresponding Author

*h.miguez@csic.es, *giovanni.bottari@uam.es.

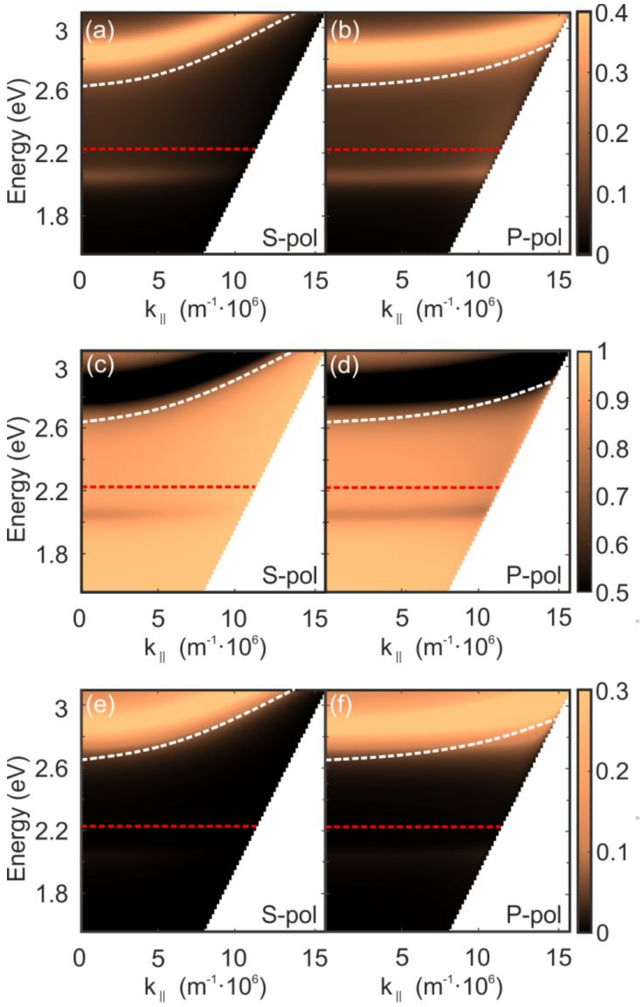

Figure S1. Dispersion curves attained from the angular dependence measurements of the absorptance, (a) and (b), reflectance, (c) and (d), and transmittance, (e) and (f), of an optical resonator made of a 50 nm thick SubPc-Et film (total cavity thickness 100 nm, including the thickness of the PVA leveling layers). Left and right panels correspond, respectively, to S (TE) and P (TM) polarized incident light. The white dashed line corresponds to the underlying cavity first order mode dispersion, while the red dashed horizontal line indicates the spectral position of the SubPc-Et absorption taken as reference.

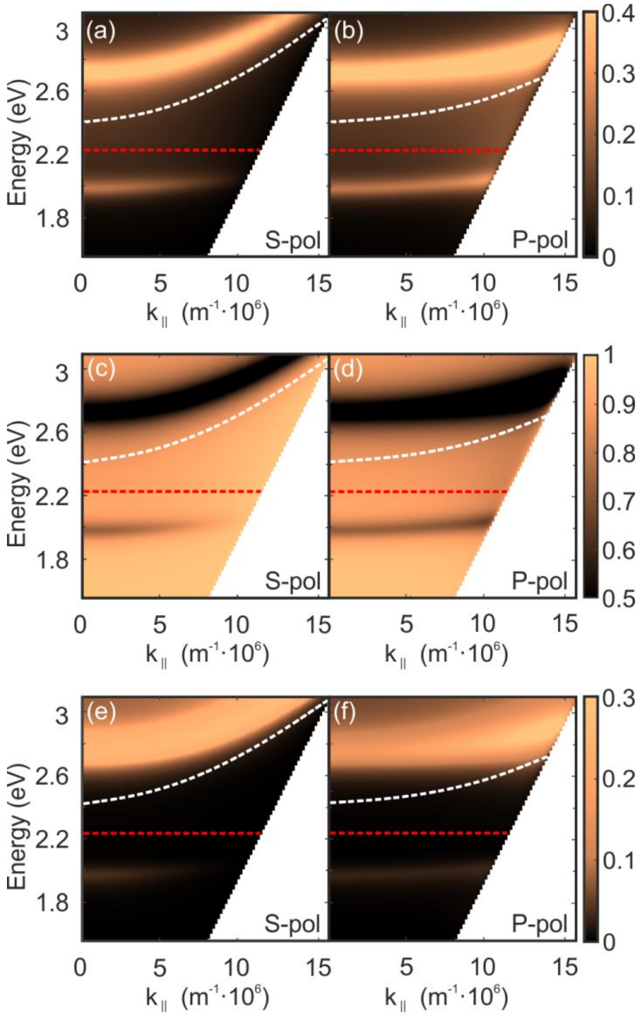

Figure S2. Dispersion curves attained from the angular dependence measurements of the absorptance, (a) and (b), reflectance, (c) and (d), and transmittance, (e) and (f), of an optical resonator made of a 71 nm thick SubPc-Et film (total cavity thickness 115 nm, including the thickness of the PVA leveling layers). Left and right panels correspond, respectively, to S (TE) and P (TM) polarized incident light. The white dashed line corresponds to the underlying cavity

first order mode dispersion, while the red dashed horizontal line indicates the spectral position of the SubPc-Et absorption taken as reference.

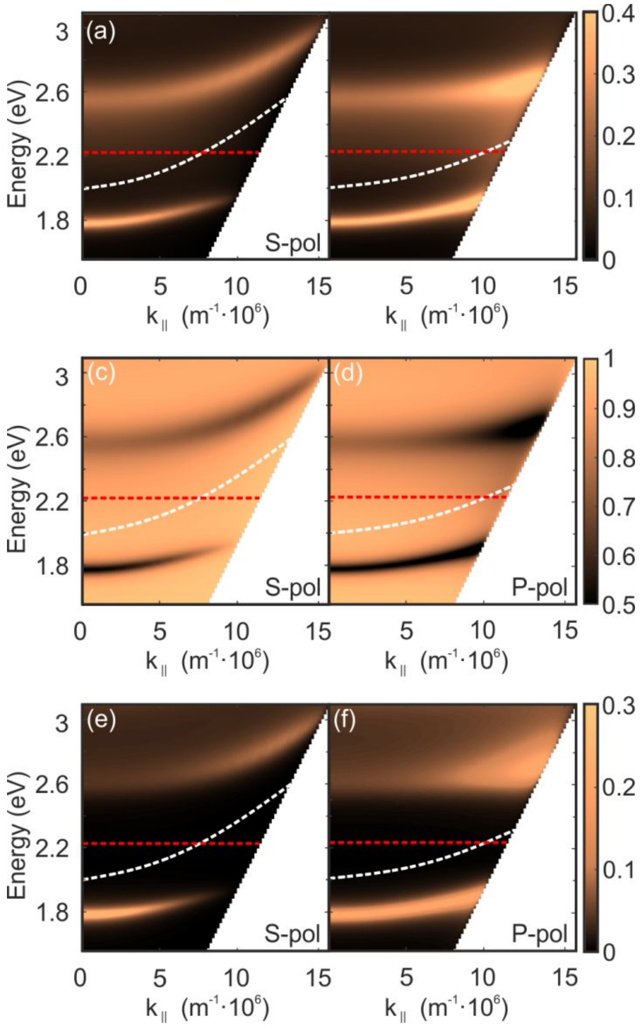

Figure S3. Dispersion curves attained from the angular dependence measurements of the absorptance, (a) and (b), reflectance, (c) and (d), and transmittance, (e) and (f), of an optical resonator made of a 106 nm thick SubPc-Et film (total cavity thickness 150 nm, including the

thickness of the PVA leveling layers). Left and right panels correspond, respectively, to S (TE) and P (TM) polarized incident light. The white dashed line corresponds to the underlying cavity first order mode dispersion, while the red dashed horizontal line indicates the spectral position of the SubPc-Et absorption taken as reference.

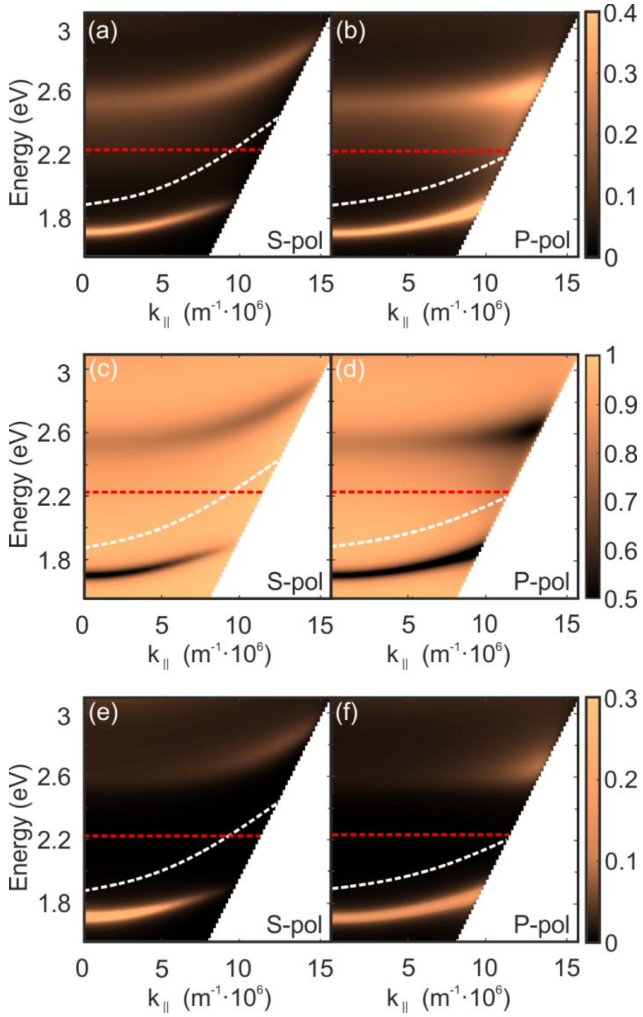

Figure S4. Dispersion curves attained from the angular dependence measurements of the absorptance, (a) and (b), reflectance, (c) and (d), and transmittance, (e) and (f), of an optical resonator made of a 113 nm thick SubPc-Et film (total cavity thickness 163 nm, including the thickness of the PVA leveling layers). Left and right panels correspond, respectively, to S (TE) and P (TM) polarized incident light. The white dashed line corresponds to the underlying cavity first order mode dispersion, while the red dashed horizontal line indicates the spectral position of the SubPc-Et absorption taken as reference.

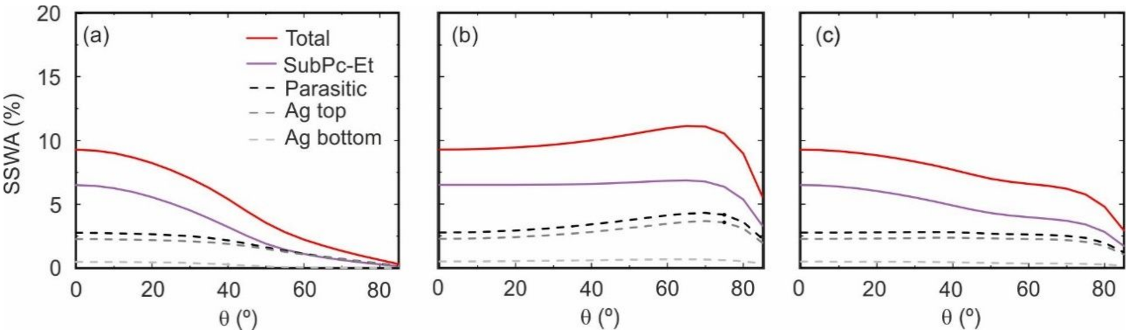

Figure S5. Wavelength integrated SSWA, versus angle of incidence of sunlight for each one of the layers in the cavity (with total cavity thickness 100 nm and δ = 0.42 eV), namely, the SubPc-Et film (purple line), top mirror (dark gray dashed line), bottom mirror (light gray dashed line). Total integrated parasitic absorption (black dashed line) and full integrated absorption of the cavity (red

solid line) are also plotted. Results are shown for (a) S-polarized, (b) P-polarized and (c) non-polarized light.

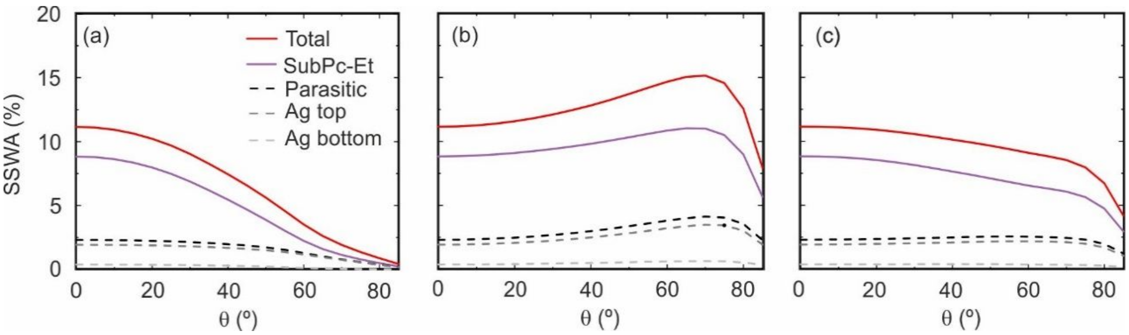

Figure S6. Wavelength integrated SSWA, versus angle of incidence of sunlight for each one of the layers in the cavity (with total cavity thickness 115 nm and δ = 0.19 eV), namely, the SubPc-Et film (purple line), top mirror (dark gray dashed line), bottom mirror (light gray dashed line). Total integrated parasitic absorption (black dashed line) and full integrated absorption of the cavity (red solid line) are also plotted. Results are shown for (a) S-polarized, (b) P-polarized and (c) non-polarized light.

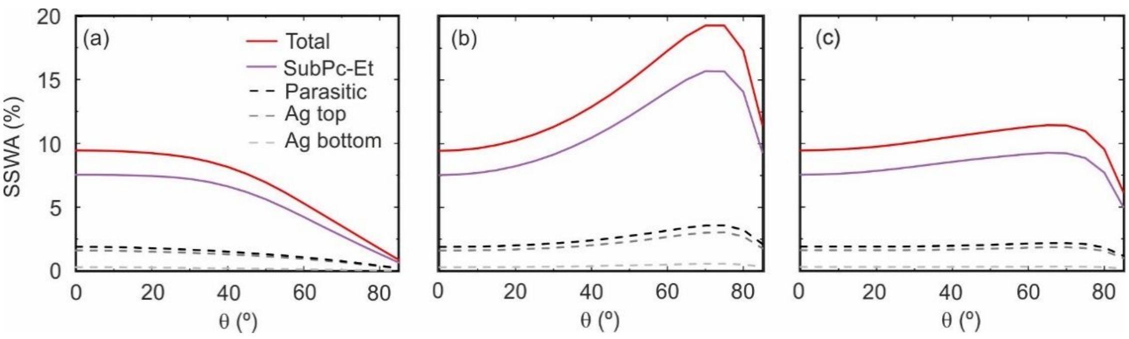

Figure S7. Wavelength integrated SSWA, versus angle of incidence of sunlight for each one of the layers in the cavity (with total cavity thickness 150 nm and δ = -0.22 eV), namely, the SubPc-Et film (purple line), top mirror (dark gray dashed line), bottom mirror (light gray dashed line). Total integrated parasitic absorption (black dashed line) and full integrated absorption of the cavity (red solid line) are also plotted. Results are shown for (a) S-polarized, (b) P-polarized and (c) non-polarized light.

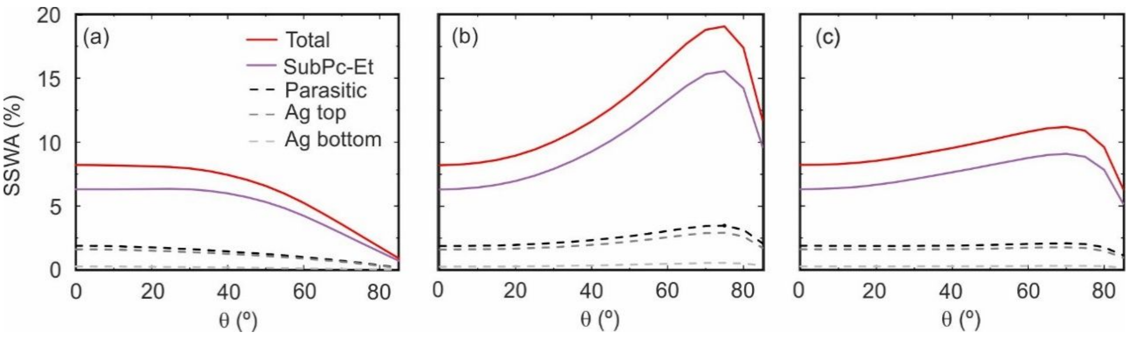

Figure S8. Wavelength integrated SSWA, versus angle of incidence of sunlight for each one of the layers in the cavity (with total cavity thickness 163 nm and δ = -0.34 eV), namely, the SubPc-Et film (purple line), top mirror (dark gray dashed line), bottom mirror (light gray dashed line). Total

integrated parasitic absorption (black dashed line) and full integrated absorption of the cavity (red solid line) are also plotted. Results are shown for (a) S-polarized, (b) P-polarized and (c) non-polarized light.

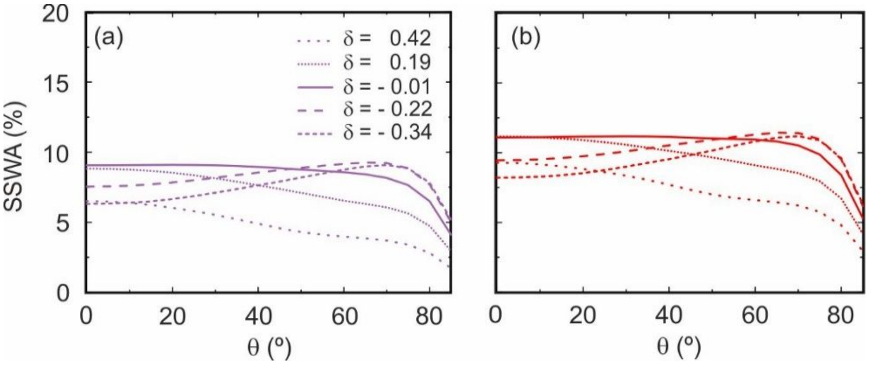

Figure S9. Wavelength integrated SSWA, versus angle of incidence of sunlight for (a) the SubPc-Et film and (b) full integrated absorption of the cavity for detuning values considered in Figure 3c. Results are shown for non-polarized light.
